# Does delocalised PCR for *Streptococcus* B in the labour ward allow adequate administration of antibiotics to prevent early neonatal infection?

**DOI:** 10.1016/j.bjid.2025.104553

**Published:** 2025-05-24

**Authors:** Clémentine Guetat, Laetitia Roussel, Marie De Antonio, Marie Accoceberry, Céline Houlle, Fanny Petillon, Marion Rouzaire, Denis Gallot

**Affiliations:** a Obstetrics and Gynaecology Department, CHU Clermont-Ferrand, France; b Biostatistics Unit, DRCI, CHU Clermont-Ferrand, France; c University Clermont Auvergne, CNRS UMR 6293, INSERM U1103, iGReD, France

**Keywords:** Group B *Streptococcus*, PCR, Pregnancy, Antibioprophylaxis, Infection

## Abstract

**Introduction:**

*Streptococcus* B is a commensal infectious agent of the intestinal and genitourinary tract. It is often implicated in early neonatal infections. Some 10 %–30 % of women are colonised by this bacterium. Screening for carriage in women before delivery prior to antibiotic prophylaxis is thus essential. In recent years, real-time PCR tests have been developed. Our main objective was to determine whether screening for *Streptococcus* B carriage by PCR on admission (gold standard GeneXpert) permits complete antibiotic prophylaxis.

**Materials and methods:**

This was an observational, retrospective study. Data set from all patients with a delocalised PCR for *Streptococcus* B (GeneXpert Instrument System) on arrival at the maternity hospital were collected between January 2022 and February 2023. We recorded 3467 test results, of which 344 were positive for *Streptococcus* B carriage. A total of 236 positive patients were included in the analysis. Antibioprophylaxis was considered complete when the patient had received at least one dose more than 4-hours before birth.

**Results:**

Of the 236 patients, antibiotic therapy was incomplete in 53 cases (22.4 %) because vaginal delivery or caesarean section occurred less than 4-hours after the first dose. Antibioprophylaxis was not initiated in 33 cases. The main reason was for rapid labour in 28 cases (11.9 %). The 5 remaining cases did not receive antibiotics because probable omission by the team (2.1 %).

**Conclusion:**

Delocalised PCR allows complete antibiotic prophylaxis against *Streptococcus* B in 63.6 % of cases, offering scope for improvement. While it will not be possible to improve antibioprophylaxis in case of rapid labour (within 3 hours after arrival), we should be able to prevent omissions (2.1 %) and, above all, reduce the birth rate before the second dose (22.4 %) by administering the first dose more quickly.

## Introduction

*Streptococcus* B (*agalactiae*) is the infectious agent most frequently incriminated in early neonatal bacterial infection. Group B *Streptococcus* is a commensal bacterium of the human intestinal and genitourinary tract, but can cause serious opportunistic infections.^1^ These bring a risk of severe morbidity that can lead to neonatal death; some 90,000 such deaths were reported by the World Health Organization in 2015.^2^ It is estimated that between 10 % and 30 % of women are colonised with *Streptococcus* B in the US and Europe.^2^, ^3^, ^4^, ^5^ Women may be colonised transiently, intermittently or persistently.

The use of intrapartum antibiotic prophylaxis in cases of maternal carriage has been shown to be effective. Conventional early culture screening between 35^+0^ and 37^+0^ WG (weeks of gestation) yields a high rate of false positives and false negatives, owing to the time lag between sampling and the actual date of delivery.^3^^,^^4^ More than 50 % of full-term infants infected with *Streptococcus* B were born to mothers who tested negative for *Streptococcus* B antepartum, prompting the search for a real-time test.^2^

For a number of years, it has been possible to carry out screening directly in early labour using PCR. GenXpert is a rapid test based on PCR technology. It uses vaginal swabs and yields a result in around 50 minutes.^6^^,^^7^ The first potential utility of an intrapartum test is to reduce unnecessary administration of antibiotics in women not requiring prophylactic treatment,^6^^,^^8^, ^9^, ^10^ thus avoiding effects on neonatal intestinal microbiota. A second utility is to ensure adequate treatment of women colonised with *Streptococcus* B, with a consequent reduction in the risk of sepsis or meningitis in the newborn.^1^^,^^7^^,^^11^

In our maternity, we adopted the exclusive use of delocalized PCR based screening for *Streptococcus* B carriage on labour admission since 2021. Compared to conventional screening based on early culture between 35^+0^ and 37^+0^ WG this strategy could delay the initiation of antibiotics due to the time required for vaginal sampling and PCR run before identification of *Streptococcus* B carriage. Our primary objective was to determine whether the exclusive use of delocalized PCR strategy at admission would enable complete antibiotic prophylaxis for *streptococcus* B carriage.

Our secondary objective was to identify the reasons for reported incomplete antibiotic prophylaxis, and whether cases of endometritis or maternal-foetal infections occurred when antibiotic prophylaxis was inadequate.

## Methods

### Population

This was an observational, retrospective, unicentric study conducted in a tertiary hospital. Data from all patients with a positive PCR for *Streptococcus* B on arrival at the maternity hospital for spontaneous labour were collected between January 2022 and February 2023.

**Fig. 1:**
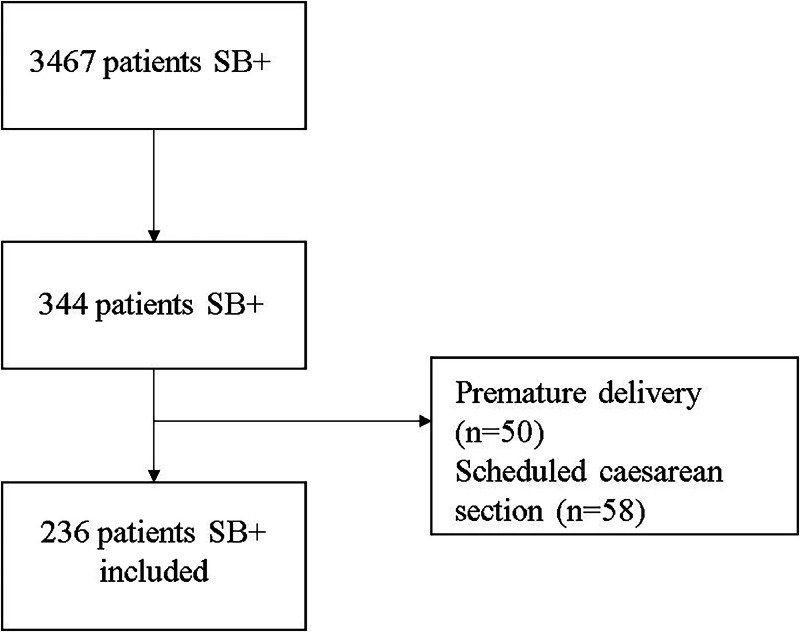
Flow-chart of the study.

We excluded patients with premature delivery because antibiotics were systematically indicated in this context and scheduled caesarean section (Fig. 1).

Clinical variables collected were age, BMI, parity, gestational age, delivery data (time of arrival at the maternity hospital, time of birth, mode of delivery, duration between rupture of membranes and birth), postpartum complications (endometritis) and neonatal characteristics such as Apgar score, weight, pH, and occurrence of infections (suspected or confirmed neonatal infection).

### Test procedure

The test was performed at admission in the labour ward by gently inserting a double swab into the patient's vagina and sampling mucosal secretions from its lower third. The swabs were rotated three times to ensure a uniform sample on both swabs.

The Xpert test was an automated *in vitro* diagnostic test for the qualitative detection of Group B *Streptococcus* (GBS) DNA. It was performed on a Cepheid GeneXpert® Instrument System (United States; Sunnyvale, California) located in the labour ward. The primers and probes of the Xpert Xpress GBS assay were designed to amplify and detect unique sequences in two chromosomal targets of GBS, one located in a coding region for a protein of the glycosyl transferase family, and the other located in a coding region for a transcriptional regulator of the LysR family of *S. agalactiae* DNA. A positive result was generated if one or both targets were detected. Results were interpreted by the GeneXpert Instrument System from measured fluorescent signals and integrated calculation algorithms.^12^ In case of initial invalid test, a second run was performed using the second swab.

### Antibioprophylaxis protocol

Antibioprophylaxis for *Streptococcal* B carriage was administered as follows:

Amoxicillin (Clamoxyl®) intravenously 2 g followed by 1 g every 4-hours from beginning of labour until delivery. In cases of allergy to penicillin, clindamycin (Dalacine®) IV 600 mg/12-hours from beginning of labour until delivery.

Antibioprophylaxis was considered complete when the patient had received at least one dose more than 4-hours before birth.^13^ We defined rapid labour as delivery within 3-hours after arrival at the maternity hospital indicating impossibility to get complete antibioprophylaxis.

### Statistics

The data are expressed as numbers and percentages n ( %) for qualitative variables, and as means (standard deviation) or medians and Interquartile range [Q1; Q3] for quantitative variables, according to statistical distribution. The assumption of normal distribution was verified using the Shapiro-Wilk test. R 4.1.3 software (*R* Foundation for Statistical Computing, Vienna, Austria) was used for analysis. As the aim of this work was mainly descriptive in order to determine whether the exclusive use of delocalized PCR strategy at admission would enable complete antibiotic prophylaxis for *Streptococcus* B carriage, no inferential statistical tests were performed.

## Results

Over the studied period, we recorded 3467 test results, of which 344 (9.9 %) were positive (Fig. 1).

After exclusion of 50 patients for premature delivery and 58 for scheduled caesarean section (not in labour), we included 236 patients.

The average age of participants was 31-years. The average BMI was 23.73 kg/m^2^.

Emergency caesarean section was performed in 12.3 % of patients, 75 % delivered by spontaneous vaginal delivery and 12.7 % delivered by instrumental vaginal delivery. Most patients arrived at the maternity hospital with intact foetal membranes (69.1 %). Population characteristics are presented in Table 1.

**Table 1:** . Demographic and obstetrical baseline data of women colonised with *Streptococcus* B included in the study.

	All (*n* = 236)
**Age (years)**	31 [28 ;35] (20 ;44)
**BMI (kg/m^2^)**	23.73 [20.78; 27.68] (14.53; 46.78)
**Parity**	2^1^^,;^^3^ (1;6)
**Mode of delivery**	
- Emergency caesarean section	29 (12.3 %)
- Spontaneous vaginal	177 (75 %)
- Instrumental	30 (12.7 %)
**Rupture of membranes (on arrival)**	73 (30.9 %)
**Duration between rupture and delivery (hours)**	5^1^^,;^^10^ (0; 97)

Data are presented as number of patients (percentages) or medians and interquartile range [Q1; Q3] (min; max) or mean (standard deviation). BMI, Body Mass Index.

Of the 236 patients, 203 (86 %) received antibiotic therapy and 150 (63.6 %) received complete antibiotic therapy (Table 2).

**Table 2:** . Implementation of antibiotic prophylaxis in SB+ patients and their infectious complications.

	All (*n* = 236)
**Antibioprophylaxis**	
- Yes	203 (86 %)
Complete	150 (63.6 %)
Incomplete	53 (22.5 %)
- Vaginal delivery < 4 h after first dose	50 (21.2 %)
- Caesarean section < 4 h after first dose	3 (1.27 %)
- No	33 (14 %)
Rapid labour (< 3 h after arrival)	28 (11.9 %)
Omission	5 (2.1 %)
**Type of antibioprophylaxis**	
- Amoxicillin	192 (94.6 %)
- Clindamycin	11 (5.4 %)
**Fever during labour**	19 (8.1 %)
**Endometritis**	3 (1.3 %)

Data are presented as number of patients (percentages).

The median time between arrival and the first dose of antibiotic was 2.73 h (1.88; 4.77). The median time between the test start and the time of the first dose of antibiotic was 1.83 h (1.3; 3.44) (including test completion time).

The median time between the first and the second dose of antibiotic was 4h 33 min (4.04; 7.83).

Antibiotic therapy was incomplete in 53 cases (22.4 %) because vaginal delivery or caesarean section occurred less than 4-hours after the first dose. Antibioprophylaxis was not initiated in 33 cases. It was associated with rapid labour (defined as delivery within 3-hours after arrival at the maternity hospital) in 28 cases, with the time between delivery and arrival at the maternity hospital ranging from 0h 02 min to 2h 12 min. The 5 remaining cases did not receive antibiotics because probable omission by the team, with times between delivery and arrival at the maternity hospital ranging from 3h to 9 h.

We noted only 1.3 % endometritis among the included patients. These patients were treated with amoxicillin + clavulanic acid. A germ was found in only one patient’s vaginal swab: it was *S. dysgalactiae*, not *Streptococcus* B. We noted that fever occurred during labour in 19 cases, which all benefited of complet antibioprophylaxis.

Paediatric data are presented in Table 3. We noted only one case of suspected maternal-foetal infection, which was treated with amoxicillin and gentamycin, with no germs found in bacteriological samples (gastric fluid and blood culture).

**Table 3:** Neonatal characteristics of children born from women colonised with *Streptococcus* B.

	**All (*n* = 236)**
**Birth weight (g)**	3317.5 (1940; 4845)
**Sex**	
- Female	126 3.4 %)
- Male	110 (46.6 %)
**Arterial pH**	7.26 [7.22; 7.3] (6.98; 7.4)
**Venous pH**	7.33 [7.29; 7.36] (7.1; 7.47)
**Lactates (mmoL/L)**	3.5 [2.5;5] (1.4;10)
**Hospitalisation**	
- Maternity unit	225 (95.3 %)
- Paediatrics	14 (5.9 %)
**Maternal-foetal infection**	1 (0.4 %)

Data are presented as number of patients (percentages) or medians and interquartile range [Q1; Q3] (min; max) or mean (standard deviation).

## Discussion

There are currently few if any studies on the optimal administration of antibiotics with the PCR test. In our study, the rate of positive tests was 10 %, in line with literature data indicating a rate of colonisation in the range 10 %–30 % in Europe. Conventional screening performed at the end of the 8th month makes the *Streptococcus* B status available before the admittance. The delocalised strategy with detection at patient admission in the labour ward could delay the initiation of administration of antibiotics due to the time required for vaginal sampling and PCR run. Our study shows that proper administration of antibiotics to reduce the risk of maternal-foetal *Streptococcus* B infections was only respected in 63.6 % of cases. Omission of treatment was observed in 2.1 % of cases and clearly requires corrective action with the aim to reduce this malpractice and its potential consequences in terms of maternal-fetal infections. Rapid delivery was associated with no antibiotics, and it concerned 11.9 % of cases. We intentionally defined rapid delivery as delivery before 3-hours after arrival. In this situation, it was clearly impossible to respect complete antibiotic administration even if conventional screening with immediate administration at arrival had been used. Median time between arrival and first antibiotic administration was 2.73-hours in our study illustrating that any delivery before 3-hours is unlikely to give the opportunity to start antibiotic administration. Moreover, in this context of rapid delivery, it is particularly challenging to collect vaginal swab, to perform PCR test and to get the screening result concomitantly with all other tasks like patient’s pain management. Incomplete administration was observed in 22.5 % of cases due to delivery less than 4-hours after the first administration. It enlightens the critical point to reduce time between arrival and first administration. The time needed to obtain the test result, approximately one hour, cannot be shortened, but the time elapsing before the test start and between the test result and the administration of the first antibiotic could be reduced. It means that care givers should make all their possible to reduce this time. We have identified two areas for improvement in terms of improving the awareness of the obstetric emergency team:

- Do the test quickly, especially for patients already in the active phase of labour;
- If the result is positive, initiate antibiotics rapidly.

This necessity should clearly be taken into account before the adoption of delocalized PCR as an unique strategy for screening of *Streptococcus* B carriers.

In the study published by El Helali et al.,^14^ it was shown that intrapartum screening for GBS by PCR was associated with a significant reduction in the rate of early-onset GBS and in the use of antibiotics in newborns. The additional costs associated with PCR were partly offset by the reduced costs of treating early-onset GBS.^15^ This further supports the use of real-time PCR.

Numerous other studies have also shown a significant reduction in the number of inadequate antimicrobial treatments, from 12 % to 4 %.^10^

In our cohort, infectious complications were rare, with three cases of endometritis and one case of maternal-foetal infection. These patients had complete antibioprophylaxis excepted one patient with endometritis, who delivered less than 4-hours after the first dose. We noted that in none of these four cases did we find any presence of *Streptococcus* B.

Despite the use of intrapartum antibiotic prophylaxis, GBS infections in newborns remain a major problem. Conventional antenatal screening for GBS carriage has shown its limitations, with some patients receiving unnecessary antibiotics, while others become colonised at the time of delivery but without receiving antibiotic therapy.

While actual recommendations considered adequate antibioprophylaxis when the patient had received at least one dose more than 4-hours before birth,^16^ revised Guidelines from CDC, 2010 stated “Shorter durations of appropriate antibiotics might provide some protection; in particular, colonization data suggest durations of ≥ 2-hours before delivery might confer some protection”.^16^ These data on the reduction in the incidence of *Streptococcus* B infections when antibiotics are administered 2-hours before delivery support the importance of starting antibioprophylaxis as early as possible, to ensure that even patients with rapid delivery receive antibiotic coverage that might confer some protection.

Real-time Polymerase Chain Reaction (PCR) can provide a result in around 50 minutes and has proved to be a specific and sensitive method for defining the status of intrapartum *Streptococcal* B carriage. The high sensitivity of real-time PCR-based tests for the identification of *S. agalactiae* has been confirmed in studies by Escobar et al.,^3^ Gerolyma et al.^1^ and Helmig et al.^17^ In this last study, the authors found a close correlation between the optimised GBS culture and the PCR test (Xpert GBS®) with a sensitivity of 100 % and a specificity of 97.5 %. Another finding of their study was the small number of invalid test results (< 1 %), although this is tempered by the fact that their swab processing and analysis were carried out in their clinical microbiology department, rather than in the delivery room.^17^ This finding was also reported in the study of Mueller et al.,^18^ who conducted a two-phase study. The first test was carried out in the laboratory and the second in the delivery room. Sensitivity was 85.7 % and specificity 95.9 % for both tests. In the laboratory phase, 8.5 % of PCR tests were found to be invalid, compared with 23.5 % in the delivery room phase, showing that the test performs better when implemented by qualified personnel.^7^ However, by processing the tests in laboratories, the time taken to obtain the results is longer, with Helmig et al. showing a delay of up to 4-hours.^4^ This encourages the test to be carried out directly in the labour ward after appropriate training of the midwives and physicians concerning vaginal sampling and use of the GenXpert system.

The data in the literature concerning the number of invalid tests is wide-ranging, from < 1 % to 23 % depending on the study.^4^^,^^7^^,^^14^^,^^17^ In our series, we observed 106 invalid tests at the first attempt but only 9/3467 (0.3 %) after using the second swab. Therefore, the risk of inadequate antibioprophylaxis induced by invalid test appears very low.

The main limitation of our study is that it was retrospective; a prospective study would provide a better level of evidence. Moreover, the single-center nature of the study means that our results cannot be generalized. To be more representative of the French population, we should have done the study in more hospitals on the territory and not only in a tertiary hospital.

Another limitation was the sample size, which makes it impossible to identify statistical significant differences between complete and incomplete antibioprophylaxis in terms of infectious risk (one case of maternal-foetal infection and three cases of endometritis). This was expected and did not compromise our ability to meet the study's objective, which was to determine whether the exclusive use of delocalized PCR strategy at admission would enable complete antibiotic prophylaxis for *Streptococcus* B carriage.

## Conclusion

Our study has highlighted a scope for improvement in the implementation of antibiotic prophylaxis for women colonised with *Streptococcus* B. While it will not be possible to improve antibioprophylaxis in case of rapid labour (within 3-hours after arrival), we should be able to prevent omissions (2.1 %) and, above all, reduce the birth rate before the second dose (22.4 %) by administering the first dose more quickly.

## Abbreviations

PCR, Polymerase Chain Reaction; WG, Weeks of Gestation; GBS, Group B *Streptococcus*; DNA, Deoxyribonucleic Acid; BMI, Body Mass Index.

## Ethics approval and consent to participate

The study was approved by our local Ethics Committee (IRB00013412, CHU de Clermont-Ferrand IRB #1, IRB number 2022-CF051) compliant with the French policy of individual data protection. Our study was retrospective research based on existing health data, and so informed consent was not required.

## Consent for publication

Not applicable.

## Author contributions

DG, CG and LR developed the study concept, design and aims, CG and LR collected the data, MDA conducted analysis of the results. CG, MDA, MR, CH, FP, MA and DG drafted and revised the paper.

## Funding

This research received no external funding.

## Conflicts of interest

Authors declare that they have no known competing financial interests or personal relationships that could have appeared to influence the work reported in this paper.

## Data Availability

The datasets used and analysed in this study are available from the corresponding author on request.
